# Estimating age-specific vaccine effectiveness using data from a large measles outbreak in Berlin, Germany, 2014/15: evidence for waning immunity

**DOI:** 10.2807/1560-7917.ES.2019.24.17.1800529

**Published:** 2019-04-25

**Authors:** Julia Bitzegeio, Shannon Majowicz, Dorothea Matysiak-Klose, Daniel Sagebiel, Dirk Werber

**Affiliations:** 1 State Office for Health and Social Affairs, Berlin, Germany; 2 Berlin School of Public Health, Berlin, Germany; 3 School of Public Health and Health Systems, University of Waterloo, Waterloo, Canada; 4 Robert Koch-Institute (RKI), Berlin, Germany

**Keywords:** measles, measles vaccine, vaccine effectiveness, waning immunity, screening method, monte carlo method, Germany

## Abstract

**Background:**

Measles elimination is based on 95% coverage with two doses of a measles-containing vaccine (MCV2), high vaccine effectiveness (VE) and life-long vaccine-induced immunity. Longitudinal analysis of antibody titres suggests existence of waning immunity, but the relevance at the population-level is unknown.

**Aim:**

We sought to assess presence of waning immunity by estimating MCV2 VE in different age groups (2–5, 6–15, 16–23, 24–30 and 31–42 years) in Berlin.

**Methods:**

We conducted a systematic literature review on vaccination coverage and applied the screening-method using data from a large measles outbreak (2014/15) in Berlin. Uncertainty in input variables was incorporated by Monte Carlo simulation. In a scenario analysis, we estimated the proportion vaccinated with MCV2 in those 31-42 years using VE of the youngest age group, where natural immunity was deemed negligible.

**Results:**

Of 773 measles cases (median age: 20 years), 40 had received MCV2. Average vaccine coverage per age group varied (32%–88%). Estimated median VE was  > 99% (95% credible interval (CrI): 98.6–100) in the three youngest age groups, but lower (90.9%, 95% CrI: 74.1–97.6) in the oldest age group. In the scenario analysis, the estimated proportion vaccinated was 98.8% (95% CrI: 96.5–99.8).

**Conclusion:**

VE for MCV2 was generally high, but lower in those aged 31-42 years old. The estimated proportion with MCV2 should have led to sufficient herd immunity in those aged 31-42 years old. Thus, lower VE cannot be fully explained by natural immunity, suggesting presence of waning immunity.

## Introduction

Measles is among the most highly transmissible infectious diseases known to affect humans and can lead to severe complications, such as pneumonia or post-infection measles encephalitis [1]. A prior infection with measles leads to life-long immunity, however, vaccination is the safest means of protection against measles. In 2012, the World Health Assembly endorsed the Global Vaccine Action Plan with the objective to eliminate measles in five of six World Health Organization (WHO) regions by 2020 [2]. Germany and the WHO European Region have committed to this goal [3,4]. Elimination is defined as the absence of endemic transmission in a country or defined geographical region for more than 36 months under a well-performing surveillance system [5]. Mathematical modelling indicates that a population immunity of up to 94% (via natural immunity or via vaccination) is necessary to reach herd immunity sufficient for elimination [6]. In the WHO European Region, the indicators for measuring progress towards measles elimination are vaccination coverage and measles incidence. The goal is to maintain at least 95% coverage with two doses of a measles-containing vaccine (MCV) at a national level and a measles incidence of less than one case per million population [3].

Since the introduction of case-based measles surveillance in Germany in 2001, the target goals for elimination were not met [7]. In Berlin, the capital of Germany with around 3.6 million inhabitants and a high population density, high incidences (range 5-145 cases per million population) have been observed from 2001 through 2013. The largest measles outbreak occurred from October 2014 to August 2015 and included a total of 1,344 measles cases in all 12 districts of Berlin, with an attack rate of 309 cases per million population [8].

Vaccination with a single dose of MCV (MCV1) was introduced in Germany in the early 1970s, followed with a two-dose scheme (MCV2) introduced in 1983 in the former German Democratic Republic and 1991 in the reunited Germany [9]. Since 2001, the standing committee on vaccination in Germany recommends the first immunisation with MCV between 11 and 14 months and the second immunisation between 15 and 23 months. Since 2010, a catch-up vaccination is recommended for all adults born after 1970 who are unvaccinated, have an unknown vaccination status or have only been given a single dose of MCV in their childhood [10]. Vaccination is voluntary and covered by insurance companies. There is no central vaccination register in Germany, so vaccination coverage is estimated based on health insurance data, school entry examinations and representative studies [11].

Current vaccination strategies assume that vaccination against measles leads to life-long immunity. However, laboratory analysis of serum samples from vaccinated persons living in areas of low endemicity show that antibody titres decrease over time, a phenomenon described as waning immunity [12-14], which could eventually lead to secondary immune failure. To evaluate the impact of this phenomenon on global elimination strategies, vaccine effectiveness (VE) has to be re-evaluated in countries close to elimination, with a special focus on older age groups where waning immunity would be most pronounced. The screening method based on Farrington’s work [15] allows VE to be estimated in the field, but it requires a valid estimate of the vaccination coverage in the general population. Thus, it is mostly used in countries with a central vaccination registry.

The main objective of our study was to estimate age-specific VE for MCV2 and investigate the presence of waning immunity, by applying the screening method on data from a large measles outbreak that occurred in Berlin 2014/15. In addition, we aimed at quantifying uncertainty in VE-estimates and the underlying input parameters, e.g. proportion of the population vaccinated (PPV) to inform further research.

## Methods

We modelled vaccine effectiveness by use of the screening method, an observational study type that uses the entire population as the reference for the proportion of the population vaccinated. The input parameters came from a retrospective outbreak analysis and from a literature review. Input parameters were described as distributions to accommodate for imperfect knowledge of the true parameters and modelled using Monte Carlo simulation.

### Data sources

#### Vaccination status of measles cases

We used notification data from a large measles outbreak in Berlin 2014/15 [8] and included all confirmed measles cases among the resident population of Berlin with a known vaccination status. Vaccination dose and date of last vaccination, as marked in the notification database, were obtained by local health authorities via vaccination records or attending physicians. Prior to analysis, entries for twice-vaccinated cases (fully vaccinated) were reviewed for accuracy with local health authorities. To exclude post-exposure vaccinations (≤ 14 days before disease onset) the time between last vaccination dose and disease onset was calculated.

Data editing and descriptive analysis of case characteristics was performed with STATA 13.1 (StataCorp LLC, Texas, United States). We calculated proportions or median values and interquartile ranges (IQR) as appropriate and conducted further analyses separately for five different age groups (2–5, 6–15, 16–23, 24–30 and 31–42 years).

#### Vaccination coverage

In this study, we determined vaccination coverage of children younger than 6 years of age using data from the association of statutory health insurance physicians [16]. Vaccination coverage of children aged 6–15 years was determined using data from school entrance health examinations that are published annually in the national bulletin for infectious diseases of the Robert Koch-Institute (RKI) [17]. For the two-dose scheme of all age cohorts older than 15 years, we conducted a systematic literature search for representative surveys. The National Center for Biotechnology Information database ‘PubMed’ was searched using the terms ‘vaccination coverage’, ‘measles’ and ‘Germany’. In addition, internal databases on the RKI website were searched for publications about vaccination coverage in the German adult population. Studies included in the analysis were any with a target population older than 15 years, representative of the target population and differentiated between the number of vaccination doses.

#### Vaccine effectiveness estimation

Age groups were constructed based on plausibility and data availability. The lower limit of 2 years was chosen because vaccination with MCV2 is recommended to be completed shortly before 2 years of age. An upper limit of 42 years was chosen due to lack of data on dose-specific vaccination coverage for the population older than 42 years. The age group 2–5 years is covered by insurance claims data and the age group 6–15 by school entry exams. For persons aged 23 and younger two doses of MCV were recommended in Germany since 1991 and for persons younger than 31 years of age two doses were recommended in the former German Democratic Republic since 1983.

VE of MCV2 was estimated with the screening method [15], which compares the vaccination coverage of cases (proportion of cases vaccinated (PCV)) with that of the general population (proportion of the population vaccinated (PPV)) using the formula VE = 1-((PCV/(1-PCV))*(1-PPV)/PPV). Dose-specific values for the PPV with two doses (PPV2) and the PCV with two doses (PCV2) were calculated as described elsewhere [18]. Briefly, PCV2 and PPV2 were calculated using only MCV2 and unvaccinated individuals, i.e. excluding individuals with MCV1. Point-estimates of PCV were computed from data from the outbreak described above. Point estimates for PPV for each age group were averaged over single years’ estimates and weighted with the relative population size of the single age cohort in Berlin [19]. If more than one result was available for an age cohort, studies were weighted equally and the average was used in the calculation. To display the uncertainty about the correct estimate, PPV was represented as a PERT distribution, a parametric distribution that has been adapted to model expert opinions by requiring only a minimum, maximum and most likely value. The minimum and maximum values in the distribution were chosen by the lowest and highest values from the single estimates within a specific age group. PCV was represented as a beta distribution. Uncertainty of the two input variables (PCV and PPV) was incorporated into the final VE estimates by performing Monte Carlo simulation with 10,000 iterations employing Monte Carlo sampling; 95% credible intervals (95% CrI) were calculated. The software ModelRisk (Vose Sint-Amandsberg, Belgium) was used for analysis.

The screening method assumes absence of natural immunity because its presence would reduce the proportion of unvaccinated susceptible persons and thus lead to an underestimation of PPV and consequently VE. The extent of natural immunity in the adult population of Berlin is unknown but assumed to be present. We aimed to evaluate the likelihood that natural immunity exclusively explains the reduced VE in the oldest age group (31–42 years), if waning immunity is excluded as contributing factor. Therefore, we conducted a scenario analysis by estimating PPV2 in this age group using the estimated distribution of VE from the youngest age group, where we deemed the presence of natural immunity to be negligible. If natural immunity would be the only contributing factor to a lower estimation for VE, the computed proportion susceptible needed to be large enough (i.e. PPV2 must be small enough) to encompass a natural immune population and a truly susceptible population in which measles could have spread (i.e. >5%, ignoring susceptible population stemming from imperfect VE of MCV1). To this end, the equation of the screening method was solved for PPV: PPV = PCV/(1 - VE + PCV*VE); Monte Carlo simulation was used as described above. 

### Ethical statement

Within the framework of the German Infection Protection Act, the State Office for Health electronically receives de-identified data on cases of notifiable infectious diseases from local health authorities. Thus, a review by an ethics committee was not required.

## Results

During the 2014/15 measles outbreak in Berlin [8], a total of 1,344 measles cases were notified. Of those, 773 cases (428 male and 345 female; age range 2–42 years) from the residential population with known vaccination status were included in the analysis in five different age groups. (Table 1). Post exposure vaccinations (MCV1: n = 32, MCV2: n = 3) were subtracted from the total number of vaccinations received, leaving 679 measles cases (87.8%) with no vaccination, 42 (5.4%) with MCV1, 40 (5.2%) with MCV2 and 12 (1.6%) with unknown vaccination dose. The proportion of cases with MCV2 was higher for those aged 23 years and older. Vaccination dates were provided for 65% of cases with MCV2. Double checking of vaccination dates and doses together with the LHA revealed no erroneous inputs.

**Table 1:** Number of measles cases in the residential population with known vaccination status during a large outbreak, by age group and vaccination status, Berlin, October 2014–August 2015

Age group (years)	Vaccinated								Unvaccinated		Total
	MCV1		MCV2		Unknown number of doses		Total				
	n	%	n	%	n	%	n	%	n	%	n
2–5	6	6.98	2	2.33	1	1.16	9	10.47	77	89.53	86
6–15	1	0.52	5	2.59	4	2.07	10	5.18	183	94.82	193
16–23	6	3.49	7	4.07	1	0.58	14	8.14	158	91.86	172
24–30	11	7.05	13	8.33	2	1.28	26	16.67	130	83.33	156
31–42	18	10.84	13	7.83	4	2.41	35	21.08	131	78.92	166
Total	42	5.43	40	5.17	12	1.55	94	12.16	679	87.84	773

MCV: measles-containing vaccine.

Ages included are 2–42 years.

The median time span since last vaccination (Table 2) indicated that most cases were vaccinated during childhood as recommended. Due to this, we used age as a proxy for the time since last vaccination.

**Table 2:** Time since last vaccination for twice-vaccinated measles cases, by age group, Berlin, October 2014–August 2015

Age group (years)	Number of cases with MCV2	Number of cases with vaccination date	Median years between disease onset and second vaccination (IQR)
2–5	2	2	3 (2.8–3.2)
6–15	5	3	8.7 (2.4–10.9)
16–23	7	5	12.5 (12.2–15.7)
24–30	13	9	24.1 (23.1–27)
31–42	13	7	30.5 (27.9–30.6)
**Total**	**40**	**26**	**21.6 (12.2–27.9)**

IQR: Interquartile range; MCV: measles-containing vaccine.

The literature search for vaccination coverage with MCV in Germany resulted in 76 hits of which, 66 were excluded as they did not address vaccination coverage for MCV or referred to an already-included study. We identified 10 studies, which we reviewed in detail – two studies were excluded as the study population was too young, one was excluded for not differentiating between number of vaccination doses and four were excluded for an unrepresentative study population (e.g. healthcare workers or medical students). In total, three studies were included in the final model [20-22]. Knowledge gaps for vaccination coverage in the population older than 15 years, especially concerning dose-specific information, were identified. Estimates for these groups relied on representative surveys that were collected in other states of Germany [20,22] or for the entire country [21], thus they were not specific to Berlin.

**Figure 1:**
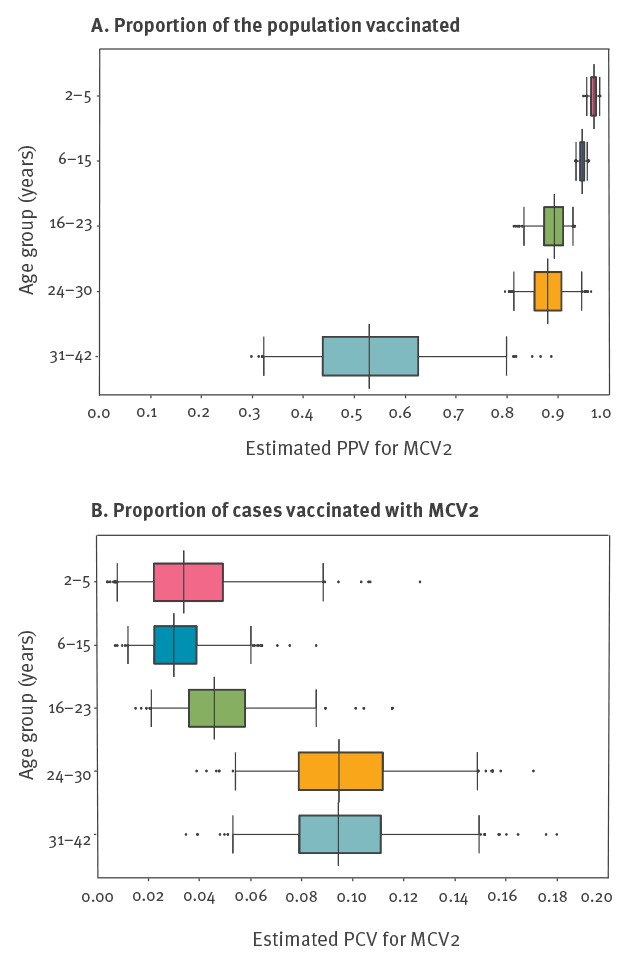
Probability distributions representing the estimated (A) proportion of the population vaccinated and (B) proportion of cases vaccinated with MCV2, by age group, Berlin, October 2014–August 2015

MCV: measles-containing vaccine; PCV: proportion of cases vaccinated; PPV: proportion of the population vaccinated.

Estimated vaccine coverage for MCV2 by age group varied from 32–88% (Table 3). The PPV2 values in the age groups under 16 years were similar, had narrow 95% Crls (2–5 years: 95% CrI: 96–98 and 6-15 years: 95% CrI: 93–96) and median values were above 94% (Figure 1A). The 95% CrI of PPV2 distributions for the age groups 24–30 years and 31–42 years were wider (95% Crl: 81–95 and 33–80, respectively), indicating greater uncertainty. PCV should positively correlate with vaccine coverage provided that the effect of natural immunity is constant across age groups and that vaccine-induced immunity does not wane. As already suggested from the raw data (Table 1), PCV2 point estimates were higher in the age groups above 24 years (Figure 1B) with a median PCV2 of 9.4% (95% CrI: 5.4–14.8) compared to the younger age groups with a median PCV2 below 5% (95% CrI: 0.8–8.8). 

**Table 3:** Data source and type for determination of vaccination coverage, vaccination coverage and proportion of the population vaccinated, by age, Berlin, 2014

Age group (years)	Data source and type for determination of vaccination coverage						Vaccination coverage			Proportion of the population vaccinated		
	Age at 2014	Age at examination (years)	Year of examination	Region	Data source	Data type	MCV1	MCV2	MCV2 average	PPV1	PPV2	PPV2 average
2–5	2	2	2016	Berlin	[16]	ASHIP	0.237	0.723	0.848	0.856	0.948	0.972
	3	3	2016	Berlin	[16]	ASHIP	0.120	0.858		0.845	0.975	
	4	4	2017	Berlin	[16]	ASHIP	0.079	0.906		0.840	0.984	
	5	5	2017	Berlin	[16]	ASHIP	0.072	0.913		0.828	0.984	
6–15	6	6	2014	Berlin	[17]	SEE	0.047	0.916	0.881	0.560	0.961	0.949
	7	6	2013	Berlin	[17]	SEE	0.052	0.908		0.565	0.958	
	8	6	2012	Berlin	[17]	SEE	0.050	0.909		0.549	0.957	
	9	6	2011	Berlin	[17]	SEE	0.052	0.907		0.559	0.957	
	10	6	2010	Berlin	[17]	SEE	0.057	0.897		0.553	0.951	
	11	6	2009	Berlin	[17]	SEE	0.060	0.891		0.550	0.948	
	12	6	2008	Berlin	[17]	SEE	0.070	0.882		0.593	0.948	
	13	6	2007	Berlin	[17]	SEE	0.077	0.868		0.583	0.940	
	14	6	2006	Berlin	[17]	SEE	0.102	0.836		0.622	0.931	
	15	6	2005	Berlin	[17]	SEE	0.147	0.788		0.693	0.924	
	15–18	7–10	2006	Germany	[21]	Survey	0.166	0.780		0.755	0.935	
16–23	15–18	7–10	2006	Germany	[21]	Survey	0.166	0.780	0.755	0.755	0.935	0.905
	19–21	11–13	2006	Germany	[21]	Survey	0.182	0.756		0.746	0.924	
	21–22	18–19	2011	Schleswig-Holstein	[22]	Survey	0.083	0.771		0.362	0.841	
	22–25	14–17	2006	Germany	[21]	Survey	0.165	0.775		0.733	0.928	
	23–32	20–29	2011	Schleswig-Holstein	[22]	Survey	0.249	0.589		0.606	0.784	
24–30	22–25	14–17	2006	Germany	[21]	Survey	0.165	0.775	0.645	0.733	0.928	0.881
	23–32	20–29	2011	Schleswig-Holstein	[22]	Survey	0.249	0.589		0.606	0.784	
	24–28	22–26	2012	Rhineland-Palatinate	[20]	Survey	0.235	0.745		0.922	0.974	
	29–33	30–31	2012	Rhineland-Palatinate	[20]	Survey	0.450	0.525		0.947	0.955	
31–42	23–32	20–29	2011	Schleswig-Holstein	[22]	Survey	0.249	0.589	0.318	0.606	0.784	0.502
	29–33	30–31	2012	Rhineland-Palatinate	[20]	Survey	0.450	0.525		0.947	0.955	
	33–42	30–39	2011	Schleswig-Holstein	[22]	Survey	0.238	0.200		0.742	0.679	
	34–38	32–36	2012	Rhineland-Palatinate	[20]	Survey	0.480	0.353		0.298	0.262	

ASHIP: association of statutory health insurance physicians; MCV: measles-containing vaccine; PPV: proportion of the population vaccinated; SEE: school entry exam.

Boxes represent the interquartile range. The median is marked by a black line and the whiskers represent the range from p2.5 to p97.5. Outliers are marked as black points.

**Figure 2:**
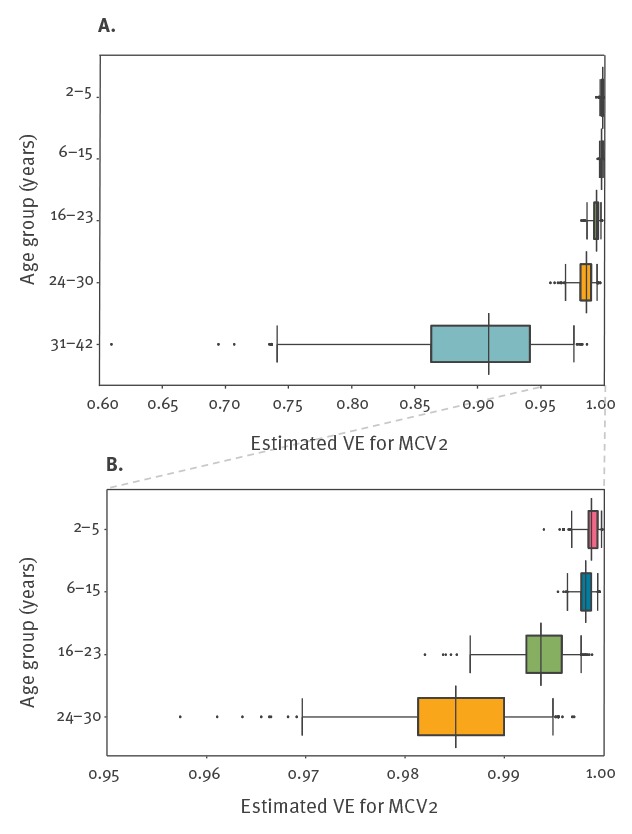
Estimated vaccine effectiveness for MCV2, by age group, Berlin, October 2014 to August 2015

MCV: measles-containing vaccine; VE: vaccine effectiveness.

The resulting distributions representing VE estimates for the five age groups showed a decline in VE with increasing age. VE estimates in the three younger age groups (2–23 years) were all above 99% (Figure 2). The estimated VE in the age group 24–30 years was slightly lower with a median of 98.5% (95% CrI: 97–99.5). The estimate for the oldest age group (31–42 years) was lower with a median of 90.9% (95% CrI: 74.1–97.6).

Boxes represent the interquartile range. The median is marked by a black line and the whiskers represent the range from p2.5 to p97.5. Outliers are marked as black points. Panel A depicts all five age groups; panel B depicts a more detailed view of only the first four age groups.

**Figure 3:**
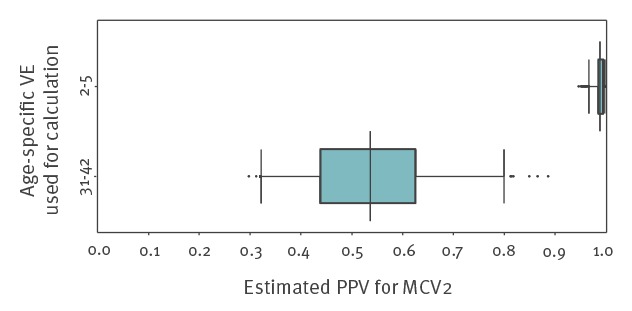
Comparison of PPV for the age group 31–42 years using VE as determined through literature review and VE estimated for age-group 2–5 years, Berlin, October 2014–August 2015

MCV: measles-containing vaccine; PPV: proportion of the population vaccinated; VE: vaccine effectiveness.

In a scenario analysis evaluating the possible influence of natural immunity, the estimated PPV for the age group 31–42 years was substantially higher (median: 98.8%; 95% CrI: 96.5–99.8) than estimated from the literature review (median: 52.9%; 95% CrI: 33.2–79.9) (Figure 3).

Boxes represent the interquartile range. The median is marked by a black line and the whiskers represent the range from p2.5 to p97.5. Outliers are marked as black points.

## Discussion

Using notification data from a large measles outbreak in Berlin in 2014/15, we estimated VE for MCV2 in different age groups to investigate signs of waning immunity in the population of Berlin. The existence of such an effect at the population level could have implications for vaccination recommendations and future elimination strategies.

A high median VE of over 99% for those aged 2–23 years for MCV2 was estimated with low uncertainty in this study. In a literature review, VE across studies has been averaged to be 94.1% [23]. Notification data capture only a fraction of actual cases occurring in the population, likely disproportionately severe cases and those with a typical clinical presentation. Patients who have been vaccinated usually present with milder and fewer symptoms and are therefore presumably under-ascertained in notification data. This may have resulted in an underestimation of PCV and an overestimation of VE in this study. When re-analysing the data, assuming a fourfold under-ascertainment of PCV, VE was still greater than 99% in the two youngest age groups (2–5 years and 6–15 years, data not shown), suggesting that the biasing effect of under-ascertained modified measles is limited. A case–control study performed in a different region in Germany during a large school outbreak in 2006 [24] (also included in the literature review) resulted in a VE estimate for MCV2 of more than 99% in persons aged 10–21 years. To reach required herd immunity of more than 94% [6], such a high VE would be necessary to actually enable elimination under the proviso of a 95% two-dose coverage strategy as recommended by WHO.

We observed a reduction of VE with increasing age and a markedly lower VE in persons 31–42 years of age, which could be a sign for waning immunity. Our results are consistent with previously published modelling approaches based on serology, that estimated the mean duration of vaccine-induced protection by MCV1 to be 25 years in the absence of re-exposure [25]. However, VE estimation by the screening method is affected by the proportion of prior cases (i.e. those with natural immunity) in the population under analysis, especially if the cases are distributed unevenly across age groups [26]. Measles is notifiable in Germany since 2001 and no reliable data have been published about the proportion of people with natural immunity in different age cohorts; this is particularly important for cohorts born before 2001 as this population showed signs of waning immunity in our study.

Before 1990, measles mortality, which can be used to estimate measles incidence, decreased gradually over time in the former Federal Republic of Germany [9]. It is assumed that the proportion of the population with natural immunity is higher in older age groups, potentially leading to an underestimation of VE, as there are less susceptible individuals in the population, which would result in an underestimation of the true PPV. Therefore, it remains unclear whether the reduced VE in the oldest age group is a result of vaccine-induced waning immunity, a high proportion of natural immunity or a mixture of both. Assuming that VE was constant with 99% across all age groups i.e. no waning immunity, we would have expected to observe no more than one measles case (instead we observed 14 cases) with MCV2 in the oldest age group, which would result in a decrease of measles cases in this outbreak by around 1%. To evaluate the potential presence of waning immunity we chose an indirect approach by computing PPV under the assumption that natural immunity would be the sole cause for reduced VE. PPV values cannot be directly translated into vaccination coverage without further knowledge about the coverage with all other doses. A value of almost 99% would indicate a very small susceptible population in the age group 31–42 years and, if this was true, measles should not have been able to spread in this population. Taking the population with only one dose of MCV into consideration, the susceptible population would be even smaller than estimated in the scenario analysis. The large number of cases in this age group and the high median age of 17 years in this outbreak, however, contradict the very low proportion of susceptible people in the oldest age group. Therefore, even though natural immunity might have influenced our VE estimates in older age groups, it is insufficient to explain the full reduction of VE in this age group.

Vaccination registries are viewed as the gold standard to monitor vaccination coverage [27]. In Germany without such registries, there was a big discrepancy between the quality of data for vaccination coverage in younger and older age groups in this study. Even though data from statutory health insurance and school entry exams gave a very detailed picture of vaccination coverage in children (2-15 years), vaccination coverage data for those older than 15 years was scarce. The data specific for vaccination doses in the population older than 24 years were derived from a telephone survey, which was carried out in 2012 in Rhineland-Palatinate [20] and a study carried out during routine occupational health checks in Schleswig-Holstein in 2011 [22] (both rural areas non-adjacent to Berlin). It is not clear how well these regions represent the multicultural and metropolitan environment of Berlin with its many temporary visitors and long-term immigrants (this would also be insufficiently covered in a central vaccination registry). In addition, the two studies yielded very different estimates for vaccination coverage in the adult population. The resulting uncertainty, especially for those aged 31–42 years, is reflected in the broad distributions for PPV.

Combining the screening method with Monte Carlo simulation, allowing the incorporation of uncertainties, may become an attractive alternative for countries that do not have a central vaccination registry, such as Germany. It allows to directly identify and quantify knowledge gaps (e.g. for vaccination coverage in certain age groups) and the model can be easily adapted to the current level of knowledge.

Aside from the potential under-ascertainment of modified measles mentioned above, our study is subject to at least two limitations. First, the screening method assumes absence of natural immunity, which is unrealistic in the population studied particularly in the older age groups; this may have resulted in an underestimation of VE in the older age groups. Although we investigated the effect indirectly in a scenario analysis, we were not able to quantify the proportion of naturally immune in the adult population to adjust our VE estimates. A serosurvey, which additionally assesses the vaccination status of the participants, could be an important add-on to obtain a reliable estimate about the immune status of the population. Second, vaccination dates were available for only 65% of all twice-vaccinated cases and only for seven of 13 cases in the oldest age group. Misclassification of vaccinated and unvaccinated cases could influence estimates for VE. Re-analysing the data for the oldest age group with only seven instead of 13 MCV2 cases we obtained an estimate for VE of 95% (data not shown), which is higher but still markedly lower than in the other four age groups.

Results of our population-based study, in keeping with serological studies [12-14], suggest the existence of a waning immunity of the measles vaccine. Although measles cases have gradually declined globally since the 1980s together with an increase in vaccination coverage [1], there has been a resurgence of measles in the European Union and European Economic Area starting in 2017 with adults aged ≥20 years comprising more than a third of all cases [28]. The impact of waning immunity to measles will likely become more apparent over the coming years and may increase in the future, as the vaccinated population (with hardly any exposure to measles) will grow older and the time since vaccination increases. It is worth noting that the median age of measles cases has been increasing over the past 15 years in Berlin [8] and the extent of waning immunity may increase further. Vaccinated cases have a lower viraemia and have rarely been observed to contribute to transmission [29,30]. However, with the vaccinated population turning older and titres possibly decreasing further, this observation has to be re-evaluated. This is of note, as the age group showing signs of waning immunity comprises individuals likely to have a young family with children, who may be too young for vaccination. In order to eliminate measles it will be crucial to regularly measure VE with a special focus on adults.

Increasing population mobility will necessitate a regular assessment of the immune status, particularly of the adult population, even in countries with a vaccination registry. The differentiation between natural and waning immunity and their effect on VE will provide information for the development of future vaccination recommendations. In response to an increase in measles cases in twice-vaccinated individuals, Taiwan now recommends an additional dose of measles vaccine for healthcare workers and aircraft staff despite high immunisation rates with two doses [31].

In conclusion, although we detected signs of waning immunity in the population of Berlin, we do not believe that vaccination recommendations should be altered at this point. Our data suggest that only a small percentage (maximum 1%) of cases could be ascribed to waning immunity and such cases are unlikely to contribute to further spread. The methodology presented here, which incorporates uncertainty into the screening method, provides a useful tool to monitor VE in countries getting closer to measles elimination with well-vaccinated birth cohorts growing older.
